# Vacuum assisted birth and risk for cerebral complications in term newborn infants: a population-based cohort study

**DOI:** 10.1186/1471-2393-14-36

**Published:** 2014-01-20

**Authors:** Cecilia Ekéus, Ulf Högberg, Mikael Norman

**Affiliations:** 1 Department of Women’s and Children’s Health, Division of Reproductive Health, Karolinska Institutet, Stockholm, Sweden; 2 Department of Women’s and Children’s Health, Uppsala University, Uppsala, Sweden; 3 Department of Clinical Science, Intervention and Technology, Division of Pediatrics, Karolinska Institutet, Stockholm, Sweden

## Abstract

**Background:**

Few studies have focused on cerebral complications among newborn infants delivered by vacuum extraction (VE). The aim of this study was to determine the risk for intracranial haemorrhage and/or cerebral dysfunction in newborn infants delivered by VE and to compare this risk with that after cesarean section in labour (CS) and spontaneous vaginal delivery, respectively.

**Methods:**

Data was obtained from Swedish national registers. In a population-based cohort from 1999 to 2010 including all singleton newborn infants delivered at term after onset of labour by VE (n = 87,150), CS (75,216) or spontaneous vaginal delivery (n = 851,347), we compared the odds for neonatal intracranial haemorrhage, traumatic or non-traumatic, convulsions or encephalopathy. Logistic regressions were used to calculate adjusted (for major risk factors and indication) odds ratios (AOR), using spontaneous vaginal delivery as reference group.

**Results:**

The rates of traumatic and non-traumatic intracranial hemorrhages were 0.8/10,000 and 3.8/1,000. VE deliveries provided 58% and 31.5% of the traumatic and non-traumatic cases, giving a ten-fold risk [AOR 10.05 (4.67-21.65)] and double risk [AOR 2.23 (1.57-3.16)], respectively. High birth weight and short mother were associated with the highest risks. Infants delivered by CS had no increased risk for intracranial hemorrhages. The risks for convulsions or encephalopathy were similar among infants delivered by VE and CS, exceeding the OR after non-assisted spontaneous vaginal delivery by two-to-three times.

**Conclusion:**

Vacuum assisted delivery is associated with increased risk for neonatal intracranial hemorrhages. Although causality could not be established in this observational study, it is important to be aware of the increased risk of intracranial hemorrhages in VE deliveries, particularly in short women and large infants. The results warrant further studies in decision making and conduct of assisted vaginal delivery.

## Background

Delivery by vacuum extraction (VE) is a common obstetrical procedure in the western world, and in many countries, it has replaced the use of forceps. The use of VE has increased from 6% in 1980 to 8.8% in all deliveries in Sweden 2011, while the use of forceps currently is 0.2% [1] In the US, vacuum-assisted births have declined to 2.8% of the births in 2011 [2].

While extra-cranial haematomas and skull fractures have been associated with VE assisted deliveries [3-7], a causal link to neonatal intracranial haemorrhage (intracranial hemorrhages;subarachnoid, subdural, and intracerebral) is less evident [8]. VE is reported to be associated with rare but severe cerebral complications [9], although study limitations have been small sample size and retrospective design [9,10], composite outcomes [11], mixed term and preterm deliveries [12,13], no comparisons of rates of intracranial complications in vacuum extraction and caesarean section (CS) deliveries [9,13]. In addition, few studies have investigated the association between VE and neonatal encephalopathy and the results are contradictive [13,14].

Intracranial hemorrhage in newborn infants can be observed also without a difficult delivery, and its complexity in etiology was already described a century ago [15]. Modern neuroimaging techniques—such as ultrasound, computerized tomography (CT), and magnetic resonance imaging (MRI)—have improved the diagnostic accuracy of neonatal brain damage. MRI in a small clinical series of asymptomatic newborn infants has revealed a high prevalence and high spontaneous resolution of small intracranial hemorrhages in both spontaneous and assisted vaginal births [16-19]. The specific risk for serious intracranial hemorrhages in relation to VE remains however to be clarified.

Sweden with one of the highest rates of VE and lowest rates of CS is well suited for a population-based cohort study comparing the risks for neonatal intracranial hemorrhages and cerebral dysfunction among term newborn infants in relation to mode of delivery. The aim of this study was to determine the risks for intracranial hemorrhages or cerebral dysfunction in newborn infants delivered by VE and to compare these risks with those after cesarean section in labour (CS) and spontaneous vaginal delivery, respectively. Furthermore, a second aim was to determine any selective contribution of mode of delivery – apart from other maternal and infant risk factors – to neonatal brain injury.

## Methods

This study was based on information in two national databases held by the Swedish National Board of Health and Welfare, and Statistics Sweden: (A) The Swedish Medical Birth Register includes prospectively collected information on demographic data, reproductive history and complications during pregnancy, delivery and the neonatal period for more than 98% of all births in Sweden. Using each mother’s unique national registration number, it is possible to link information on successive births within the Medical Birth Register and to link information between registries. Maternal characteristics are recorded in a standardized manner during a woman’s first visit to antenatal care, which occurs before 15 weeks of gestation in more than 95% of the pregnancies and (B) The Swedish National Inpatient Register, which covers all public in-patient care. The national registration number, assigned to each Swedish resident at birth, was used for individual record linkage.

The study population was retrieved from the Swedish Medical Birth Register and included all singleton newborn infants in Sweden between 1999 and 2010 delivered at term (gestational age >37 weeks + 0 days) after the onset of labour by vacuum extraction VE (in all VE n = 87,150, including failed VE ending in CS n = 3484) by cesarean section in labour CS (n = 75,216), or by spontaneous vaginal delivery (n = 851,347). Stillborn infants, multiple births, infants delivered by elective CS before labour, breech deliveries and forceps-assisted deliveries were excluded. Since the use of forceps has declined from 0.5% in 1999 to 0.2% in 2010 and now only constitutes a fraction of all deliveries in Sweden, we decided to exclude this mode of delivery in this study. Thus, the study population included 94% of all deliveries among term, singleton, live-born infants during the study period.

Information about parity (primi- or multipara), maternal age, height, body mass index (BMI), and mode of delivery was collected from the The Swedish Medical Birth Register. BMI was calculated from measured height and weight, obtained from the first antenatal care visit at 8–10 gestational weeks and categorized into underweight (below 18.5 kg/m^2^), normal (18.5–24.9), overweight (25–29.9), obese (>29.9), or missing. CS was defined as abdominal delivery after the onset of labour. Gestational age (GA: categorized into 37–38, 39–41, and 42–45 weeks) was recorded in completed weeks, and was based on routine ultrasound dating performed at 17 to 18 postmenstrual weeks in 97–98% of all pregnant women. Indications for VE and CS were classified into prolonged labour (O62.0-2, O63.0-9), signs of fetal distress (O68.0-O68.1-9), a combination of these, or none of these using obstetric diagnoses—collected from the Swedish Medical Birth Register or the Swedish National Inpatient Register —classified according to the International Classification of Diseases (ICD) tenth edition (1997 and onwards) revisions.

The following ICD-10 codes were assessed as outcomes: intracranial laceration and haemorrhage due to birth injury (P10), intracranial non-traumatic haemorrhage of foetus and newborn (P52), convulsions of newborn (P90), and other disturbances of the cerebral status of the newborn; encephalopathy (P91). The definition of each outcome is described in detail in Table 1. Infants that had at least one outcome diagnosis in The Swedish Medical Birth Register or in the Swedish National Inpatient Register were counted as cases. More than 85% of the outcome diagnoses were retrieved from the Swedish Medical Birth Register and 15% came from the Swedish National Inpatient Register. The registers do not cover information on when an infant was diagnosed.

**Table 1:** Neonatal outcomes studied in term, singleton newborn infants

**Neonatal outcomes**			
**Outcome**	**Main ICD-code**	**ICD-subgroup**	
**Intracranial bleeding**	P10 Intracranial laceration and haemorrhage due to birth injury	10.0	Subdural haemorrhage due to birth injury
		10.1	Cerebral haemorrhage due to birth injury
		10.2	Intraventricular haemorrhage due to birth injury
		10.3	Subarachnoid haemorrhage due to birth injury
		10.4	Tentorial tear due to birth injury
		10.8	Other intracranial lacerations and haemorrhages due to birth injury
		10.9	Unspecified intracranial laceration and haemorrhage due to birth injury
	P52 Intracranial non-traumatic haemorrhage of foetus and newborn	52.0	Intraventricular (non-traumatic) haemorrhage, grade 1, Subependymal haemorrhage (without intraventricular extension)
		52.1	Intraventricular (non-traumatic) haemorrhage, grade 2, Subependymal haemorrhage with intraventricular extension
		52.2	Intraventricular (non-traumatic) haemorrhage, grade 3,
			Subependymal haemorrhage with both intraventricular and intracerebral extension
		52.3	Unspecified intraventricular (non-traumatic) haemorrhage of foetus and newborn
		52.4	Intracerebral (non-traumatic) haemorrhage of fetus and newborn
		52.5	Subarachnoid (non-traumatic) haemorrhage of foetus and newborn
		52.6	Cerebella (non-traumatic) and posterior fossa haemorrhage of fetus and newborn
		52.8	Other intracranial (non-traumatic) haemorrhages of foetus and newborn
		52.9	Intracranial (non-traumatic) haemorrhage of foetus and newborn, unspecified
**Neonatal cerebral dysfunction**	P 90 Convulsions of newborn		
	P91 Other disturbances of cerebral status of newborn/Encephalopathy	P91.0	Neonatal cerebral ischemia
		P91.1	Acquired periventricular cysts of newborn
		P91.2	Neonatal cerebral leukomalacia
		P91.3	Neonatal cerebral irritability
		P91.4	Neonatal cerebral depression
		P91.5	Neonatal coma
		P91.6	Hypoxic ischemic encephalopathy of newborn
		P91.8	Other specified disturbances of cerebral status of newborn
		P91.9	Disturbance of cerebral status of newborn, unspecified

During the study period, neonatal diagnoses of an intracranial lesion were based on imaging of the brain using ultrasonography, CT, and/or MRI. During the study period, MRI was introduced and to some extent replaced CT for neonatal brain imaging. The rate of intracranial hemorrages did not change significantly, however, in relation to year of birth. Imaging of the brain was performed on clinical indications in all cases and there was no screening—general or selective, based on risk factors—of asymptomatic infants. A diagnosis of convulsions included infants with clinical signs of convulsions and/or convulsions verified by EEG.

Statistical analysis was performed using proportions and odds ratios (OR) with a 95% confidence interval (CI) for severe neonatal cerebral complications in relation to mode of delivery, using spontaneous vaginal delivery as the reference group (SPSS 20.0 for Windows software package). Three models were used to assess the relationship between the different modes of delivery and the risk for neonatal cerebral complications, one crude and two adjusted (Models 1 and 2). The included co-variates have been shown previously to be related to instrumental deliveries and were related to the outcomes in cross tabulations [20-22]. In Model 1, we adjusted for the following confounders or co-variates: year of birth; parity; maternal age, height, and BMI; and infant birthweight and GA. In Model 2, we added shoulder dystocia and the indication for operative delivery. The year of birth was entered as a continuous variable in accordance with a linear secular trend, and all other variables were entered as categories. In the adjusted model, we refrained from stratifying by hospital type or by annual number of deliveries due to the fact that outcomes were overall rare and each strata would have contained only very limited or no numbers. Missing data were entered as a separate category in the analyses. The study was approved by the Regional Ethical Review Board in Stockholm, Dnr 2008/1322-31.

## Results

During the study period, the proportion of women delivered by VE was on average of 8.6% with an annual variation from 7.6 to 9.1%, and by CS (in labour), 7.4%, with an annual variation from 6.6% to 7.9%. The rate of VE varied between 6.2% to 13.4% between hospitals, and the rate of CS varied from 6.1% to 11.0%. The numbers of newborn infants with any cerebral complication delivered by VE was 906 (104/10,000) and by CS the numbers were 652 (87/10,000) compared with 1,227 (14/10,000) after spontaneous vaginal delivery.

The rate of newborn infants with intracranial hemorrhages was 4.9/10,000 in university hospitals and 3.8/10,000 in county hospitals. The corresponding rates for encephalopaties/convulsions were 23.9 and 25.0, respectively. The differences between university and county hospitals were not statistically significant.

### Maternal and perinatal characteristics by mode of delivery

Primiparas were overrepresented among women delivered by VE and CS, while multiparas were overrepresented among women delivering vaginally without operative assistance. In the CS group, more infants were post-term (GA 42–45 weeks), and more women were overweight or obese as compared to women in the VE and vaginal delivery groups; see Table 2.

**Table 2:** Maternal and perinatal characteristics by mode of delivery in a population-based cohort of singleton pregnancies starting with labour and ending at term

	**Spontaneous vaginal**	**Emergency CS**	**Vacuum extraction**
**N = 1,010,229**	**N = 851,347**	**N = 75,216**	**N = 87,150**
	%	%	%
**Maternal age (years)**			
-19	1.9	1.1	1.6
20-24	13.6	10.1	13.5
25-29	31.2	28.1	32.8
30-34	34.6	36.1	34.8
35-39	15.6	19.4	14.4
>39	2.8	4.7	2.6
Missing	0.3	0.4	0.3
**Maternal height (cm)**			
-155	3.6	8.1	4.7
156-160	12.8	19.4	15.2
161-165	24.7	27.1	26.1
166-170	28.5	23.9	26.9
>170	24.5	15.0	20.9
Missing	6.0	6.4	6.1
**Maternal BMI**			
Underweight	1.5	0.9	1.6
Normal	36.6	30.3	38.1
Overweight	14.3	17.6	14.2
Obese	5.8	10.0	5.0
Missing	41.8	41.2	41.1
**Parity**			
Multipara	60.7	35.3	20.8
Primipara	39.3	64.7	79.2
**Indication**			
Signs of fetal distress	1.1	29.7	34.9
Prolonged labour	6.4	32.9	38.3
Both	0.2	8.3	10.8
None of these	92.3	29.2	16.1
**Gestational week**			
37-38	16.3	16.8	11.4
39-41	77.1	64.3	75.8
42-45	6.6	18.9	12.8
**Infant birthweight (g)**			
≤3000	9.5	11.1	9.2
3001-3500	32.9	25.5	30.9
3501-4000	37.7	33.0	37.7
4001-4500	16.1	21.6	17.9
>4500 gram	3.6	8.6	4.1
Missing	0.2	0.3	0.2

### Neonatal intracranial haemorrhage by mode of delivery

In all, 86 newborn infants were diagnosed with intracranial laceration and haemorrhage classified as traumatic intracranial hemorrhages), corresponding to a rate of 0.8/10,000 births, and 384 infants were diagnosed with non-traumatic intracranial hemorrhages), corresponding to a rate of 3.8/10,000 births. Eight infants had both diagnoses. Among the infants diagnosed with traumatic intracranial hemorrhages, 58% were delivered by VE, 7.1% with CS and 35% by spontaneous vaginal delivery. Among those diagnosed with non-traumatic intracranial hemorrhages, the corresponding proportions were 32%, 13%, and 56%, respectively, for each mode of delivery.

The rate of neonatal intracranial hemorrhages (both traumatic and non-traumatic intracranial hemorrhages) was more than six times greater among newborns delivered by VE (19.0 per 10,000) and more than doubled among those born by CS (7.3 per 10,000) compared with infants born by spontaneous vaginal delivery (2.8 per 10,000). Intracranial hemorrhages were generally more frequent among infants of primiparas than of multiparas women. Among VE-delivered infants, the rate of intracranial hemorrhages increased gradually with increasing birthweight (except infants with a birthweight below 3000 gram), increasing maternal BMI, and decreasing maternal height. Infants diagnosed with shoulder dystocia had the highest rates, 131/10,000, Table 3.

**Table 3:** Frequencies and crude rates of neonatal intracranial haemorrhage (diagnoses P10 and P52) in term singleton infants categorized by mode of delivery

**N= 1,010,229**	**Traumatic and non-traumatic intracranial haemorrhage of fetus and newborn n = 462**								
		**Vaginal delivery**			**Emergency cesarean section**			**Vacuum extraction**	
		**N =851,347**			**N = 75,216**			**N = 87,150**	
	**N**	**n**	**1/10 000**	**N**	**n**	**1/10 000**	**N**	**n**	**1/10 000**
**Total**	**851,347**	**241**	**2.8**	**75,216**	**55**	**7.3**	**87,150**	**166**	**19.0**
**Maternal age (years)**									
-19	16,256	4	2.5	859	0		1,384	1	7.2
20-34	676,207	177	2.6	55,934	44	7.9	70,645	137	19.4
<34	156,454	59	3.8	18,151	11	6.1	14,820	28	18.9
Missing	2,430	1	4.1	272	0		301	0	
**Maternal height (cm)**									
-155	30,767	14	4.6	6,116	7	11.4	4,074	18	44.2
156-160	108,658	28	2.6	14,622	12	8.2	13,278	31	23.3
161-165	210,094	68	3.2	20,403	10	4.9	22,733	38	16.7
166-170	242,617	67	2.8	17,965	11	6.1	23,470	39	16.6
>170	208,213	53	2.5	11,276	8	7.1	18,240	24	13.2
**Missing**	50,998	11	2.2	4,834	7	14.5	5,355	16	29.9
**Maternal BMI**									
Underweight	12,843	4	3.1	643	0		1,372	1	7.3
Normal	311,523	68	2.2	22,768	11	4.8	33,207	62	18.7
Overweight	121,733	42	3.5	13,246	10	7.5	12,395	24	19.4
Obese	49,278	22	4.5	7543	9	11.9	4,369	8	18.3
Missing	355,970	105	2.9	31,016	25	8.1	35,807	71	19.8
**Parity**									
Multipara	516,619	101	2.0	26,521	16	6.0	18,091	26	14.4
Primipara	334,728	140	4.2	48,695	39	8.0	69,059	140	20.3
**Indication**									
Signs of fetal distress	9,403	10	10.6	22,320	38	17.0	22,320	57	18.8
Prolonged labour	54,204	28	5.2	24,738	8	3.2	24,738	64	19.2
Both	1,834	2	10.9	6,225	2	3.2	6,225	22	23.4
None of these	785,906	201	2.6	21,933	7	3.2	21,933	23	16.4
**Gestational week**									
37-38	138,934	66	4.8	12,641	14	11.1	9,949	24	24.1
39-41	656,094	154	2.3	48,343	32	6.6	66,020	111	16.8
42-45	56,319	21	3.7	14,232	9	6.3	11,181	31	27.7
**Infant birthweight (g)**									
≤3000	60,674	44	5.5	8,334	14	16.8	7,985	13	16.3
3001-3500	280,255	69	2.5	19,163	9	4.7	26,914	38	14.1
3501-4000	320,650	69	2.2	24,807	18	7.3	32,870	63	19.2
4001-4500	136,803	42	3.1	16,236	7	4.3	15,596	35	22.4
>4500	30,861	17	5.5	6,455	2	3.1	3,571	14	39.2
Missing	2,104	0		221	5	226.2	214	3	140.2
**Shoulder dystocia**									
No	849,910	240	2.8	75,212	55	7.3	86,310	155	18.0
Yes	1,437	1	7.0	4	0		840	11	131.0

### Neonatal convulsions and encephalopathy by mode of delivery

In all, 1,763 newborn infants were diagnosed with convulsions and 1,629 with encephalopathy), 583 infants had both these diagnoses.

Infants delivered by CS or VE had six-to-seven times higher rates of convulsions or encephalopathy than those born by spontaneous vaginal delivery. The rate increased with increasing maternal BMI in all types of delivery, and with decreasing maternal height, particularly in the VE-group. In the VE-group, increasing infant birthweight was gradually related to neonatal convulsions or encephalopathy, whereas in the CS-group this relationship was inversely related. Finally, the rate of convulsions or encephalopathy was almost doubled in VE-delivered infants born after 41 weeks of GA as compared to those born in weeks 39–41; see Table 4.

**Table 4:** Frequencies and crude rates of convulsions and other disturbances of cerebral function (ICD10 diagnoses P90 and P91) in term singleton infants categorized by mode of delivery

**N= 1,010,229**	**Convulsions or encephalopathy n = 2,587**								
		**Vaginal delivery**			**Emergency cesarean section**			**Vacuum extraction**	
		**N =851,347**			**N =75,216**			**N = 87,150**	
	**N**	**n**	**1/10,000**	**N**	**n**	**1/10 000**	**N**	**n**	**1/10 000**
**Total**	**851,347**	**1,113**	**13.1**	**75,216**	**627**	**83.4**	**87,150**	**847**	**97.2**
**Maternal age (years)**									
-19	16,256	16	9.8	859	6	69.8	1,384	11	79.5
20-34	676,207	875	12.9	55,934	457	81.7	70,645	673	95.3
>34	156,454	217	13.9	18,151	160	88.1	14,820	161	108.6
Missing	2,430	5	20.6	272	4	147.1	301	2	66.4
**Maternal height (cm)**									
-155	30,767	52	16.9	6,116	46	75.2	4,074	65	159.5
156-160	108,658	167	15.4	14,622	120	82.1	13,278	174	131.0
161-165	210,094	313	14.9	20,403	143	70.1	22,733	233	102.5
166-170	242,617	281	11.6	17,965	155	86.3	23,470	194	82.7
>170	208,213	224	10.8	11,276	107	94.9	18,240	131	71.8
Missing	50,998	76	14.9	4,834	56	115.8	5,355	50	93.4
**Maternal BMI**									
Underweight	12,843	9	7.0	643	3	46.7	1,372	6	43.7
Normal	311,523	343	11.0	22,768	165	72.5	33,207	288	8.67
Overweight	121,733	170	14.0	13,246	109	82.3	12,395	141	113.8
Obese	49,278	105	21.3	7,543	77	102.1	4,369	59	135.0
Missing	355,970	486	13.7	31,016	273	88.0	35,807	353	98.6
**Parity**									
Multipara	516,619	514	9.9	26,521	265	99.9	18,091	177	97.8
Primi	334,728	599	17.9	48,695	362	74.3	69,059	670	97.0
**Indication**									
Signs of fetal distress	9,403	81	86.1	22,320	415	185.9	22,320	300	102.6
Prolonged labour	54,204	140	25.8	24,738	49	19.8	24,738	158	83.5
Both	1,834	16	87.2	6,225	42	67.5	6,225	80	160.4
None of these	785,906	876	11.1	21,933	121	55.2	21,933	309	60.9
**Gestational week**									
37-38	138,934	178	12.8	12,641	94	74.4	9,949	103	103.5
39-41	656,094	807	12.3	48,343	419	86.7	66,020	581	88.0
42-45	56,319	128	22.7	14,232	114	80.1	11,181	163	145.8
**Infant birth weight (g)**									
≤3000	80,674	149	18.5	8,334	111	133.2	7,985	59	73.9
3001-3500	280,255	298	10.6	19,163	166	86.6	26,914	230	85.5
3501-4000	320,650	344	10.7	24,807	184	74.2	32,870	298	90.7
4001-4500	136,803	205	15.0	16,236	102	62.8	15,596	167	107.1
>4500	30,861	87	28.2	6,455	29	44.9	3,571	61	170.8
Missing	2,104	30	142.6	221	35	1,583.7	214	32	1495.3
**Shoulder dystocia**									
No	849,910	1,064	12.5	75,212	627	83.4	86,310	775	89.8
Yes	1,437	49	341.0	4	0		840	72	857.1

Table 5 shows crude and adjusted odds ratios for the neonatal outcomes by mode of delivery, with infants born by spontaneous vaginal delivery as the reference group. Here we present intracranial hemorrhages as two separate outcomes: intra-cranial lacerations and haemorrhage due to birth injury and, intracranial non-traumatic haemorrhage of foetus and newborn. After adjustment for indication for operative delivery and other co-variates, newborn infants delivered by VE had a ten-fold higher risk for traumatic intracranial hemorrhages and more than a doubled risk for non-traumatic intracranial hemorrhages, whereas infants delivered by CS had no increased risk for either traumatic or non-traumatic intracranial hemorrhages. Maternal characteristics, parity, GA, and birthweight (Model 1) explained 25%, and indication for instrumental delivery (Model 2), a further 21% of the observed risk increase for traumatic intracranial hemorrhages in infants delivered by VE compared to spontaneous vaginal delivery. The corresponding proportions for non-traumatic intracranial hemorrhages were 30% and 61%, respectively.

**Table 5:** Logistic regression (odds ratios: OR, crude and adjusted) for intracranial laceration and haemorrhage due to birth injury (P10), intracranial non-traumatic haemorrhage (P52), neonatal convulsions (P90) or other disturbances of cerebral status of newborn (P91) by mode of delivery

	**P 10 intracranial laceration and haemorrhage due to birth injury**					
**Mode of delivery**	**N**	**n**	**1/10 000**	**Crude OR 95% CI**	**Model 1**	**Model 2**
Vaginal	851,347	30	0.4	1.0	1.0	1.0
CS	75,216	6	0.8	2.26 (0.94-5.44)	1.43 (0.58-3.53)	1.27 (0.46-3.50)
VE	87,150	50	5.7	16.29 (10.36-25.62)	12.43 (7.58-20.38)	10.05 (4.67-21.65)
Total	1,013,713	86	0.8			
	**P 52 intracranial non-traumatic haemorrhage**					
Vaginal	851,347	214	2.5	1.0	1.0	1.0
CS	75,216	49	6.5	2.59 (1.90-3.54)	1.69 (1.22-2.35)	1.03 (0.70-1.53)
VE	87,150	121	13.9	5.53 (4.42-6.91)	4.18 (3.29-5.30)	2.23 (1.57-3.16)
Total	1,013,713	384	3.8			
	**P 90 and/or P91 convulsions and/or encephalopathy**					
Vaginal	851,347	1,113	13.1	1.0	1.0	1.0
CS	75,216	627	83.4	6.42 (5.82-7.08)	5.02 (4.52-5.58)	2.49 (2.17-2.87)
VE	87,150	847	97.2	7.50 (6.85-8.20)	6.55 (5.95-7.21)	2.61 (2.27-3.00)
Total	1,013,713	2,587	25.5			

Model 1 ORs adjusted for year of birth, maternal age, maternal height and BMI, parity, gestational age and infant birthweight.

Model 2 ORs also adjusted for shoulder dystocia indications of operative delivery (signs of foetal distress, prolonged labor or foetal distress and prolonged labor).

After adjustment for indication for operative delivery and other co-variates, newborn infants delivered by VE or CS faced more than a doubled risk for convulsions or encephalopathy as compared with infants delivered vaginally without operative assistance.

## Discussion

In this national cohort study, we found traumatic intracranial hemorrhages in 6/10,000 and of non-traumatic intracranial hemorrhages in 14/10,000 newborn infants delivered at term by VE. The ORs for intracranial hemorrhages after VE were significantly higher (ten-fold higher for traumatic and doubled for non-traumatic haemorrhage) compared with ORs found after delivery by CS and non-assisted vaginally delivery. High birthweight and a short mother were associated with the highest ORs for neonatal intracranial hemorrhages after VE. The rates of neonatal convulsions or encephalopathy were two to three times higher, but almost the same in both VE deliveries and CS. This indicates that different mechanisms are involved in the development of the two types of cerebral complications.

Our study confirms the previously described association between VE-assisted birth and increased risk for neonatal intracranial hemorrhages, and provides robust data on incidence and risk factors for this complication. The finding that VE but not CS was associated with increased risk for neonatal intracranial hemorrhages contrasts, however, to previous observations. There is only one large population-based and nowadays old (from 1992–94) study in which a relation between all types of operative delivery (VE, forceps and CS) and increased rates of neonatal intracranial hemorrhages was found. Based on these findings, the authors concluded that abnormal labour, rather than mode of delivery contributed to increased risk for intracranial injury [23].

In the present study we investigated infants admitted for neonatal care because of clinical symptoms after birth. A neonatal diagnosis of intracranial haemorrhage, convulsion, or cerebral dysfunction most likely represents the most severe degrees of these complications [24].

In VE deliveries, we found particularly high rates in of all cerebral complications among infants with high birthweight. This finding is consistent with another study [10] and indicates that extractions may become more difficult with increasing birthweight. In addition, short maternal stature and high maternal BMI were gradually associated with intracranial hemorrhages. All these factors are associated with prolonged labour and instrumental delivery [21] and might be due to a relative cephalopelvic disproportion.

Although VE was related to significantly increased rates of intracranial hemorrhages, it is not clear whether the extraction as such could cause cerebral complications or whether it is the complications that lead to the need for a VE delivery that causes intracranial hemorrhages. The axial pressure gradient to the head in labour peaks during the second stage of delivery, and few cesarean deliveries are done during second stage of labour. Thus dystocic labour that results in a delivery by vacuum or cesarean may have the same diagnosis, but certainly the infant born by vacuum-assisted delivery should have been exposed to a higher pressure (duration and force) due to labour per se.

A major strength of this study was the nationwide population-based design, allowing for accurate estimates of rare adverse events, such as severe neonatal cerebral complications of clinical relevance. We were able to include data on risk factors, potential confounders, and outcomes collected independently from one another and without involving the study subjects, thus minimizing various types of bias (e.g., selection, recall). Another advantage was the inclusion of the main indications for VE and CS, enabling us to address the question of confounding by indication. The main exposures—proportion of deliveries by VE and CS, showed homogeneity over time but varied among types of hospital. The main outcome, intracranial hemorrhages, did not differ in relation to year of birth, in either university or county hospitals.

Limitations are that we could not verify the registry-stated indication for operative delivery, and we did not have information on the severity and timing of complications indicating operative delivery. Moreover, the registry does not provide specific information about the type of VE instrument used, level, position, and attitude of the fetal head in the pelvis when applying VE, location of placement of the vacuum cup, traction work, skill of the obstetrician, pressure, exposure time and cup detachments. In addition, the register does not provide information about use of oxytocin and application of fundal pressure both increasing the axial pressure on the presenting part. Malmström, who developed the modern ventouse, showed in an experiment that applied external pressure is spread over a sphere while the pressure within the sphere increased by 6% [25], in contrast to external fundal pressure increasing the pressure gradient by 17% [26]. It might be the case that failed VE could represent worst cases of child outcome, but exclusion of those cases did not significantly change the results. Bias due the high number of missing height and BMI is not probable, neither it is a systematic missing in the the Swedish Medical Birth Register nor it is a lack of power in the study sample.

Infant diagnosis as outcome measures also might have limitations such as lack of uniform guidelines on indication for neuroimaging and diagnostic evaluation of newborn infants with clinical suspicion of central nervous dysfunction, as well as changes in neuroimaging diagnostics over time. However, the rates of intracranial hemorrhages did not differ in relation to year of birth or between university and county hospitals.

As diagnostic procedures where done on clinical indications, detection bias with underestimation of the rate of intracranial hemorrhages in the spontaneous vaginal delivered could not excluded [16-19]. However, underestimation of the true intracranial hemorrhages -rate following VE may also have occurred. In a case series of term infants (n = 913) screened with transfontanellar ultrasound after VE, the rate of intracranial homorrhages was reported to be 4.6 times higher (0.87%) than in our study [9]. In that study, most of the patients were reported to exhibit “reassuring clinical status” and only one infant with intracranial hemorrhages was admitted for neonatal intensive care.

## Conclusions

Newborn term infants delivered by VE at term have in general low but significantly higher rates of intracranial haemorrhages compared with those born by CS or by a non-assisted vaginal delivery, also after taking indications of operative delivery into account. High infant birthweight and short maternal height were associated with the highest risk for cerebral complications after VE. A cautious interpretation of these results could be awareness of the increased risk of intracranial haemorrhage in vacuum-assisted deliveries, particularly in short women expecting a large infant. However, causality has not been established and more studies are needed to disentangle whether the risks observed herein can with certainty be attributed to detection bias, inherent instrumentation, technique problems or residual confounding.

## Abbreviations

AOR: Adjusted odds ratios; BMI: Body mass index; CI: Confidence interval; CS: Cesarean section; CT: Computerized tomography; GA: Gestational age; MRI: Magnetic resonance imaging; OR: Odds ratios; VE: Vacuum extraction.

## Competing interests

There are no conflicts of interest for any of the authors. There are no financial competing interests.

## Authors’ contributions

CE had the idea for the study, designed it, carried out the statistical analysis, and wrote the first draft of the manuscript. UH and MN contributed to the interpretation of results and writing of the manuscript and approved the final version of the submitted article. All authors read and approved the final manuscript.

## Pre-publication history

The pre-publication history for this paper can be accessed here:

http://www.biomedcentral.com/1471-2393/14/36/prepub
